# Sustainable production of *Pleurotus sajor-caju* mushrooms and biocomposites using brewer’s spent and agro-industrial residues

**DOI:** 10.1038/s41598-024-77435-1

**Published:** 2024-11-01

**Authors:** Joara Lúcia do Nascimento Deschamps, João Guilherme Schulz, Josiane Costa Riani, Mariane Bonatti-Chaves, Michelle Bonatti, Stefan Sieber, Marcos Lana, Elisabeth Wisbeck

**Affiliations:** 1 Master Science in Process Engineering, University of the Region of Joinville (UNIVILLE), Rua Paulo Malshitzki, 10, 89.219-710, Joinville, SC Brazil; 2 Department of Mechanical Engineering, University of the Region of Joinville (UNIVILLE), Rua Paulo Malshitzki, 10, 89.219-710, Joinville, SC Brazil; 3 Department of Chemical Engineering, University of the Region of Joinville (UNIVILLE), Rua Paulo Malshitzki, 10, 89.219-710, Joinville, SC Brazil; 4 Master Science in Productive Systems, University of the Region of Joinville (UNIVILLE), Rua Paulo Malshitzki, 10, 89.219-710, Joinville, SC Brazil; 5 Leibniz Centre for Agricultural Landscape Research (ZALF e. V), Eberswalder Straße 84, 15374 Muncheberg, Germany; 6 Department of Agricultural Economics, Humboldt University of Berlim (HU), Invalidenstr. 42, 10115 Berlim, Germany; 7 Swedish University of Agricultural Sciences (SLU), P.O. Box 7070, Uppsala, 750 07 Sweden

**Keywords:** Brewer´s spent, Agro-industrial residues, Mycelium-based composites, *Pleurotus sajor-caju*, Sustainable production, Biotechnology, Environmental sciences

## Abstract

Brazil is one of the world’s largest beer producers and also a major food producer. These activities generate a large amount of residues which, if disposed of inappropriately, can have adverse effects on the environment. The objective of this research was to evaluate the potential of using these residues for both mushroom cultivation (traditional use) and the production of mycelium-based composites (innovative use). Mushroom production (*Pleurotus sajor-caju*) was conducted using only brewer’s spent grains (fresh and dried) and also mixed with banana leaves (1:1) or peach palm leaves (1:1), which are residues widely available in the northern region of Santa Catarina, Brazil. The productivity of mushrooms cultivated using fresh and dried brewer’s spent grains did not exhibit a statistically significant difference, indicating that this residue can be utilized shortly after its generation in the industrial process, thereby reducing costs associated with production. Combining brewer’s spent grains with banana or peach palm leaves resulted in enhanced mushroom production (0.41 and 0.38 g day^−1^, respectively) compared to using the leaves as a sole substrate. The mushrooms produced contain sugars and a minimal sodium content, and are considered a source of phosphorus. In addition, no toxic elements (Hg and Pb) were present. The mycelium-based composites produced using the residual substrate (after the mushroom harvest) exhibited better mechanical properties (compressive strength = 0.04 MPa, density = 242 kg m^−3^, and low humidity sorption) than those produced using fresh substrate. The results demonstrate the synergistic effect of combining the two approaches under investigation. The use of brewer´s spent enhance the mushroom productivity and the residual substrate enhance the mechanical properties of mycelium-based composites. The compressive strength, density, and air humidity sorption properties are essential for determining the potential applications of mycelium-based composites. The use of brewer’s spent grains mixed with banana leaves demonstrated significant promise for mushroom production and subsequent application in the development of mycelium-based composites. These sequential approaches contribute to waste valorization and the rational utilization of natural resources, as the mycelium-based composites are considered for substitution of synthetic materials, thereby promoting sustainability for future generations.

## Introduction

In 2022, global beer production reached approximately 1.89 billion hectoliters. The three largest beer producers are China (360.41 million hectoliters), the United States (194.1 million hectoliters), and Brazil (147.43 million hectoliters)^1^. According to the Kirin Global Beer Report^2^, in 2022, Brazil ranks third in global beer consumption, with 14,932 thousand kl consumed, representing 7.8% of the global market share. This high beer production has led to increased brewer’s spending, which has environmental implications. Due to the high concentration of organic material and high humidity (approximately 80%), brewer’s spent can have a negative impact on the environment when it is unduly routed to nature. In particular, the brewer’s spent grain, which constitutes approximately 85% of all byproducts generated during the brewing of beer, is often utilized in livestock diets due to its elevated protein content and relatively low cost. Furthermore, the brewer’s spent grain can be sold to producers of dried spent grain. However, in regions where the industry for breeding ruminants and other domestic animals is underdeveloped, the brewer’s spent grain is often disposed in fields or incinerated. The brewer’s spent grain represents the insoluble components that remain after the lautering process, which occurs just before fermentation. In addition to the soluble components that are attached to the grain, including maltooligomers, maltose, and glucose, the dry matter of brewer’s spent grain also includes cellulose, lignin, proteins, lipids, hemicellulose, and an ash fraction. Indeed, alternative applications are currently under investigation, including its potential use as a food supplement in the food industry, in bioremediation and recycling agricultural processes, in bioinsecticide production, bioelectricity generation, biogas, bioethanol, biodiesel, and mushrooms production^3–7^.

Mushrooms are considered healthy foods that contain a high protein content, all essential amino acids, a high proportion of unsaturated fatty acids, various vitamins and minerals, and low lipids, cholesterol, nucleic acids, and calories^8,9^. Mushrooms of the genus *Pleurotus* represent the second most popular variety for cultivation, constituting 25% of global production^9^. In Brazil, this group also occupies the second position, with its production representing an estimated 16% of the total fresh mushrooms produced in the country^10^. The variety of suitable substrates (agro-industrial residues), ease of cultivation, ease of maintenance, and high nutritional value have contributed to enhanced mushroom production in this genus on a global scale^11,12^. This has also fostered research into different biotechnological applications of the genus^5,7–9,13^ including the production of mycelial-based composites^14,15^.

At the conclusion of the mushroom harvesting process, a residue, referred to as residual substrate, is generated and may be employed in the manufacture of mycelium-based composites. The mycelium-based composite is produced by inoculating a filamentous fungus on a substrate consisting of discontinuous particles of nutrient material. The fungus will metabolize the nutrient material over a period sufficient to grow hyphae, allowing them to form an interconnected mycelial network in and around the substrate, binding the particles of material that take on the shape of the container in which they are grown. Its uses include replacing wooden, plastic, foam, and Styrofoam packaging^14^.

The use of composite materials derived from renewable natural resources, such as mycelium-based composites, is increasingly attracting attention as a sustainable alternative to the use of fossil and oil-based materials^15–18^. Concerns about environmental sustainability are pushing the use of innovative materials with diverse applications to replace conventional synthetic materials^19^. Mycelium-based composites have diverse advantages over traditional synthetic polymers, including their low density, less energy-intensive manufacturing process, and perhaps most importantly, their biodegradability and environment-friendly nature, enabling an entirely circular production model^15^. They can be used in various applications such as automobiles, aerospace, packaging and building industries, and sports instruments^20^. Biocomposites produced from fungal growth can be more efficient and cost-effective than many synthetic and organic processes due to their high level of bioefficiency and ability to use multiple nutrients and resource sources^14,15,17,18^. Among the various mechanical properties of fungal biocomposites, compressive strength^14,15,21–23^, water sorption^14,22^, and density^14,15,22^ are frequently evaluated.

The hypothesis of this study is that brewer’s spent grains, banana leaves, and peach palm leaves, which are generated in large amounts in the northeast region of Santa Catarina (Brazil), can serve as efficient substrates for the production of high-quality mushrooms. In addition, these materials can be used in the production of mycelium-based composites, either in a sub-sequential process or directly. This approach has the potential to contribute to the development of a novel process within the circular economy, whereby waste is valorized and natural resources are utilized more efficiently.

## Materials and methods

The studies of mushroom production were carried out in 2016, while the studies using it to produce mycelium-based composites were carried out in 2020. All experiments were carried out in the biotechnology laboratories of the University of the Region of Joinville, Joinville, Brazil.

### Microorganism and inoculum

*Pleurotus sajor-caju* was selected for this study due to its classification as a primary decomposer of plant residues and its natural ability to grow in tropical, subtropical and temperate regions, which facilitates the artificial cultivation of *Pleurotus* spp. Additionally, *Pleurotus* spp. is the second most consumed and commercialized mushroom in Brazil, and it is one of the most frequently referenced fungal genera in the literature for the production of mycelium-based composites^10,15^. *Pleurotus sajor-caju* was obtained from the Collection of Basidiomycetes Cultures of the Botany Institute (São Paulo-SP) under the code CCB 019. The strain was maintained in wheat dextrose agar (TDA) medium^24^ under refrigeration (4 °C). The wheat grains were cooked, supplemented with CaCO_3_ (0.35%) and CaSO_4_ (1.35%), packed (250 g of wheat grains in a 20 × 30 cm polypropylene package), sealed with foam breathers to facilitate gas exchange, and sterilized at 121 °C and 1 atm for 1 h. After sterilization, they were inoculated with three 8 mm diameter agar discs containing mycelium and incubated at 28 ± 2 °C in the dark until complete colonization of the surface of the grains by the mycelium occurred after 15 days^6^.

## Preparation of substrates

The substrates for the production of mycelium-based composites are originate from three sources: agricultural by-products, industrial waste, and post-consumer waste^23^. In this study were used the industrial waste brewer’s spent grains mixed with the agricultural by-products banana leaves and peach palm leaves. These residues are abundant in the northern region of Santa Catarina, Brazil, and were supplied by a local producer. The brewer’s spent grain was tested in two forms: in the original form (SN – natural), which was used immediately after collection from the industry, and in a stored form, after which it was dried at 60 °C (SS – dried). Before mushroom production, the SS – dried residue was rehydrated and immersed in water for 12 h, after which the excess water was drained for 2 h. However, due to high compaction after sterilization, which could make mushroom production unfeasible, SN and SS were also tested in a mixture with banana leaves (1:1) (SNB and SSB) and in a mixture with peach palm leaves (1:1) (SNP and SSP). The banana leaves and peach palm leaves (naturally dried) were cut into 2–5 cm fractions and packed in raffia bags immersed in water as described above^25^. For all six formulations of substrate (Table 1), the equivalent of 150 g of the dry substrate was packaged into a polypropylene bag (28 × 40 cm), and 5% (7.5 g) of the rice bran was added as a nitrogen source, to improve the substrate’s compatibility with the mycelium. In SN and SS, 0.35% CaCO_3_ and 1.3% CaSO_4_ were added to the polypropylene bags to avoid compaction of the substrate. All bags were then sterilized at 121 °C for 2 h in an autoclave.

**Table 1:** Substrates formulations and corresponding abbreviations.

Abbreviations	Substrate formulation
SN	Fresh brewer’s spent grains + 5% rice bran + 0.35% CaCO_3_ + 1.3% CaSO_4_
SS	Dried brewer’s spent grains + 5% rice bran + 0.35% CaCO_3_ + 1.3% CaSO_4_
SNB	Fresh brewer’s spent grains + banana leaves (1:1) + 5% rice bran
SSB	Dried brewer’s spent grains + banana leaves (1:1) + 5% rice bran
SNP	Fresh brewer’s spent grains + peach palm leaves (1:1) + 5% rice bran
SSP	Dried brewer’s spent grains + peach palm leaves (1:1) + 5% rice bran

## Mushroom production

The polypropylene package bags were inoculated in a biological safety cabinet with 20% inoculum of *P. sajor-caju* relative to the dry substrate mass (150 g) and incubated in the dark at 28 ± 2 °C until complete colonization of the substrate by fungal mycelium. The induction of the primordia was accomplished by perforating the packages and exposing them to light (270 lx) for 12 h per day with a relative air humidity of approximately 90%. The mushrooms were harvested when the edges of the pileus appeared flat in the stage prior to sporulation^26^. Mushrooms were harvested using a scalpel, weighed to determine the fresh mass, and dehydrated at 40 °C for 24 h with forced air circulation (1370fx, SHELLAB, Cornelius, USA) to determine the dried mass.

## Analyzes of the substrate and mushroom

The mushroom yield was evaluated in terms of biological efficiency (*BE -* %) (Eq. 1)^27^ and productivity (*Pr -* g day^−1^) (Eq. 2)^28^.

1 $$\:BE\left(\%\right)=\:\frac{dried\:mushrooms\:mass\:\left(g\right)}{dried\:initial\:substrate\:mass\:\left(g\right)}\times\:100$$

2 $$\:Pr\left(g\:{day}^{-1}\right)=\:\frac{dried\:mushrooms\:mass\:\left(g\right)}{dried\:substrate\:mass\:\left(g\right).\:total\:cultivation\:time\:\left(day\right)}\:\times\:\:100$$

For the chemical analyzes, 200 g of brewer’s spent grains (SN and SS) and 200 g of dried mushroom (105 °C until constant mass was reached) were used. The mushrooms selected for these nutritional and chemical analyzes were those obtained from the experiments that presented the best values of EB and Pr. The samples of brewer’s spent grain, and mushrooms were subjected to carbohydrate^29^, protein, crude fat, crude fiber, ash, and moisture^30^ analyzes. The crude protein content was calculated by multiplying the total nitrogen content by a correction factor of 6.25 for the brewer’s spent^30^ and 4.38 for the mushrooms^11^. To determine the carbon: nitrogen ratio (C:N) of the brewer’s spent grain, the organic carbon was divided by the total nitrogen content. The organic carbon content was calculated by dividing the organic matter content by the van Bemmelen conversion factor of 1.72. The potassium, sodium, lead, and mercury contents of the mushrooms were determined via atomic absorption spectrometry, and the phosphorus content was determined via UV‒VIS spectrophotometry^31^.

## Mycelium-based composite production

The first substrate evaluated was prepared using the natural brewer’s spent grains (SN – fresh) mixed with banana leaves (1:1) (called SNB fresh). Initially, the naturally dried banana leaves were cut into 2–5 cm fractions, packed in raffia bags, rehydrated in water for 12 h and then drained for approximately 2 h^25^. The fresh brewer´s spent grains were mixed with the banana leaves and an equivalent mass of 100 g of the dry substrate was packed in a polypropylene bag (28 × 40 cm), 5% (5 g) rice bran was added as a source of nitrogen. The humidity contents of the fresh brewer’s spent grains and banana leaves were 73.2 and 75.5%, respectively^28^. The second substrate evaluated was obtained from fresh brewer’s spent grains mixed with banana leaves (1:1) after harvesting the mushrooms (called SNB residual). Initially, the SNB residual was dried at 105 °C, disintegrated (crumbed) and packed in raffia bags that were immersed in water for 12 h for rehydration and then drained for approximately 2 h^25^. Thereafter, an equivalent mass of 100 g of the dry substrate was packed in a polypropylene bag (28 × 40 cm).

**Fig. 1:**
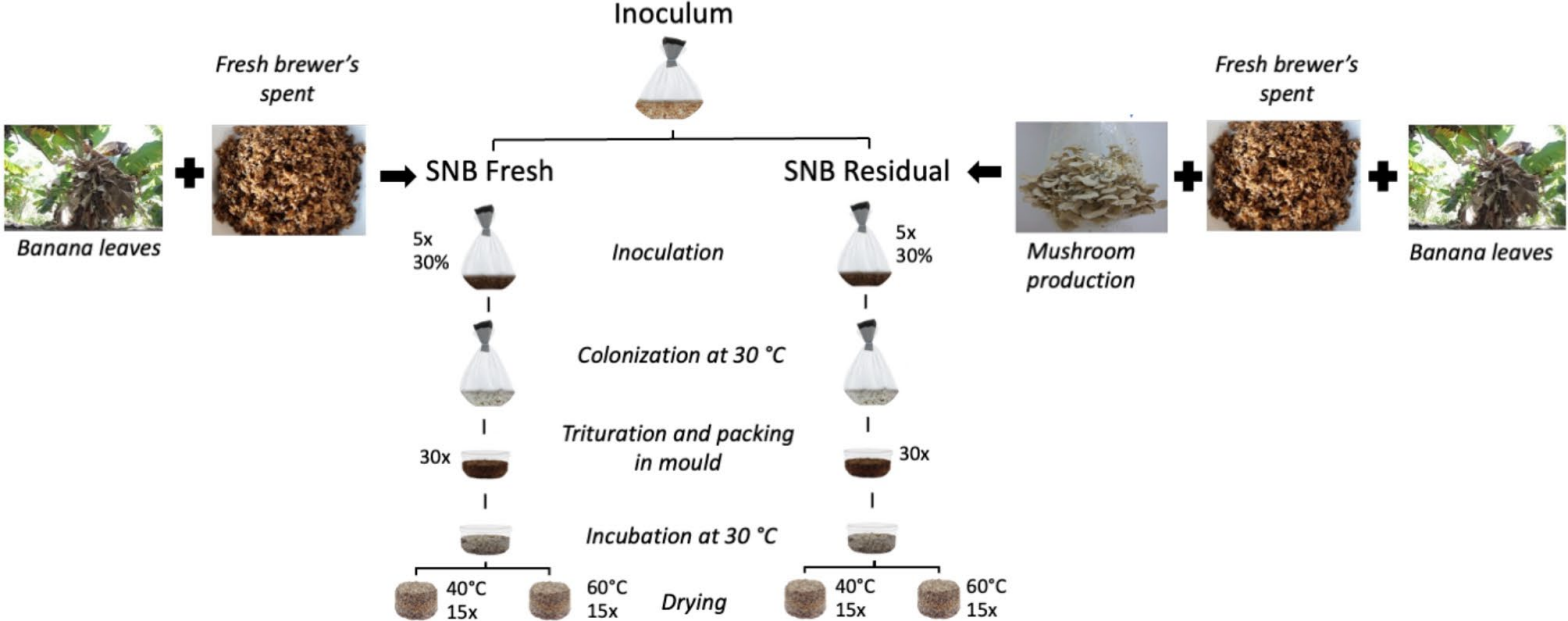
Diagram of the mycelium-based composites process manufacturing.

All bags were then sterilized at 121 °C for 2 h in an autoclave; subsequently, the substrates (SNB fresh and SNB residual) were inoculated in a biological safety cabinet (Veco VLSF-12) with the inoculum of *Pleurotus sajor-caju*, using 30% of the inoculum in relation to the dry mass of the substrate. Five packages were prepared for each type of substrate (SNB fresh and SNB residual). The packages were incubated at 30 °C in the absence of light until complete substrate colonization by fungal mycelium. The colonized substrates were then mixed (five packages for each type of substrate) and ground in a food processor until a homogeneous mixture was obtained. The homogeneous mixtures were placed in 30 cylindrical plastic moulds (6 cm diameter), 30 for each type of substrate, and aseptically compacted to a height of 2.5 cm to obtain the proof bodies^32^. The moulds were closed and incubated in the absence of light at 30 °C until the substrate was completely recolonized by the fungal mycelium. Afterwards, the mycelium-based composites were dried in an oven with forced air circulation (SHELLAB 1370 FX) at 60 °C (Fig. 1).

### Analysis of mycelium-based composites

For the characterization of the mycelium-based composites, water sorption analysis, air humidity sorption, compressive strength, apparent density, and soil biodegradability were performed. The analysis of water sorption was based on the D-570 repeated immersion method^33^. Three proof bodies were selected from each treatment, each having their initial mass (*M*_0_) measured before immersion in distilled water at a pH of 7 ± 1 and a temperature of 25 ± 1 °C, remaining covered by a column of 25 ± 5 mm of water for 2 and 24 h to verify the water sorption stabilization. To analyze the influence of air humidity, the three proof bodies from each treatment were exposed to ambient air with relative air humidity and room temperature monitored (AR-807 Digital Hygrometer Smart Sensor Temperature Humidity) every two days for 60 days, simulating the exposure of this material to the environmental conditions.

The compressive strength test was performed on EMIC DL 10,000/700 equipment according to NBR 8082^32^, with seven proof bodies from each treatment analyzed. The load cell used was 10^5^ N, and the crosspiece speed was 5 mm min^−1^. In each quadrant of the proof bodies, the thickness and diameter were measured using a caliper to obtain an average initial thickness and an average diameter for calculating the cross-sectional area. The average diameter was 5.6 mm, and the height was 2.3 mm. The compressive strength (*σc*) was calculated at 10% strain, which is the maximum strain allowed for practical use (*σc* = F·A^−1^), where (F) is the compression force and (A) is the cross-sectional area of the body proofs. To evaluate whether the time of exposure to air humidity influences the compressive strength of the mycelium-based composites, body proofs after 60 days of exposure to air humidity were also subjected to a compression test. To obtain the apparent density of the material (*d* = *m*·*v*^−1^), the proof bodies were weighed immediately after drying (40 and 60 °C), the mass (*m*) of the material was obtained, and in each specimen quadrant, the thickness and diameter were measured with a pachymeter to obtain the initial mean thickness and mean diameter, thus obtaining the volume (v = Ab x h), where Ab = base area and h = height. The analysis of biodegradability in soil followed the ‘soil burial’ methodology test according to the G160 norm^34^. Two proof bodies of each treatment were buried at a depth of approximately 10 cm in previously prepared soil and conditioned in 1 L Becker glasses with a height of approximately 17 cm. The Becker glasses were kept in a room with automatic controls of temperature (30 ± 2 °C) and a relative humidity of 85 to 95%. Visual analysis was performed after 15, 30, 60, and 120 days. At this time, the samples were removed, carefully cleaned, and photographed.

### Statistical analysis

The values obtained in the characterization of the SN and SS substrates, in the nutritional analysis of mushrooms, in the productive parameters (biological efficiency and productivity), and in the mycelium-based composite analysis were subjected to an outlier rejection test (Dixon Q test) and subsequently analyzed using variance analysis of the mean values with the Tukey test at a significance level of 5% (ANOVA).

## Results and discussion

Initially, brewer’s spent grain was characterized in terms of carbohydrates, proteins, fat, crude fiber, ash, moisture, and the carbon: nitrogen ratio (C:N) (Table 2). There was a significant difference in moisture content between the SN (73.2%) and SS (60.7%) substrates. The low moisture content found in the SS substrate is because this residue has undergone a drying process before immersion. After drying, cellulose fibers are tightly clustered, resulting in shrinkage and loss of pores in the cell wall, which irreversibly impairs the ability to absorb water^36^. However, the moisture content found in SN (73.2%) is comparable to that reported by other authors, which ranged from 70 to 80%^3,37,38^.

As illustrated in Table 2, the protein content remained unaltered following the drying of the brewer’s spent grain (SS). The contents of lipids and crude fiber increased when comparing the SN and SS, likely due to the concentration of these components following the drying of the substrate. The observed changes in the carbohydrate, ash, and C: N contents, which decreased after substrate drying, may be related to the immersion of the substrate in water. This is because a portion of the soluble solids are washed during the immersion process^39,40^.

**Table 2:** Composition (mean ± standard deviation) of brewer’s spent grain (SN: natural, unprocessed; SS: dried) on a dry basis. Equivalent letters indicate no significant difference between the SN and SS (Tukey test, 5% significance).

Composition	Mean ± standard deviation	
	SN	SS
Moisture substrate (%)	73.2 ± 0.58a	60.7 ± 1.26b
Total carbohydrates (%)	68.4 ± 0.00a	64.3 ± 0.00b
Crude protein (%)	10.3 ± 0.00a	10.4 ± 0.07a
Lipids (%)	3.9 ± 0.01a	6.1 ± 0.07b
Crude fiber (%)	13.9 ± 0.47a	16.0 ± 0.52b
Ashed (%)	3.5 ± 0.04a	3.2 ± 0.05b
Carbon: Nitrogen ratio (C:N)	(34.0: 1) ± 0.04a	(33.8: 1) ± 0.01b

Despite the reduction in total carbohydrates from 68.4% (SN) to 64.3% (SS), these values are similar to those reported by other authors^3,5,41^ in brewer’s spent grain. Regarding proteins, lower and higher values of protein than those reported in this study were found in brewer’s spent grain according to the literature (5 to 25%)^3,4,37,41^. The lipid content observed in the literature ranged from 2.5 to 10%^4,41^. The fiber content reported in this study is lower than that reported in the literature (20–70%)^41–43^. Although the ash content shows a statistically significant difference, decreasing from 3.5 to 3.2 for substrates SN to SS, these values are similar to those described in the literature^4,41–43^. In terms of the carbon-to-nitrogen ratio, the values for the SN and SS substrates were 34.0:1 and 33.8:1, respectively. These values were significantly different; however, both values were above 29:1, which is considered optimal for the growth and development of *Pleurotus* mushroom mycelia^44^.

**Fig. 2:**
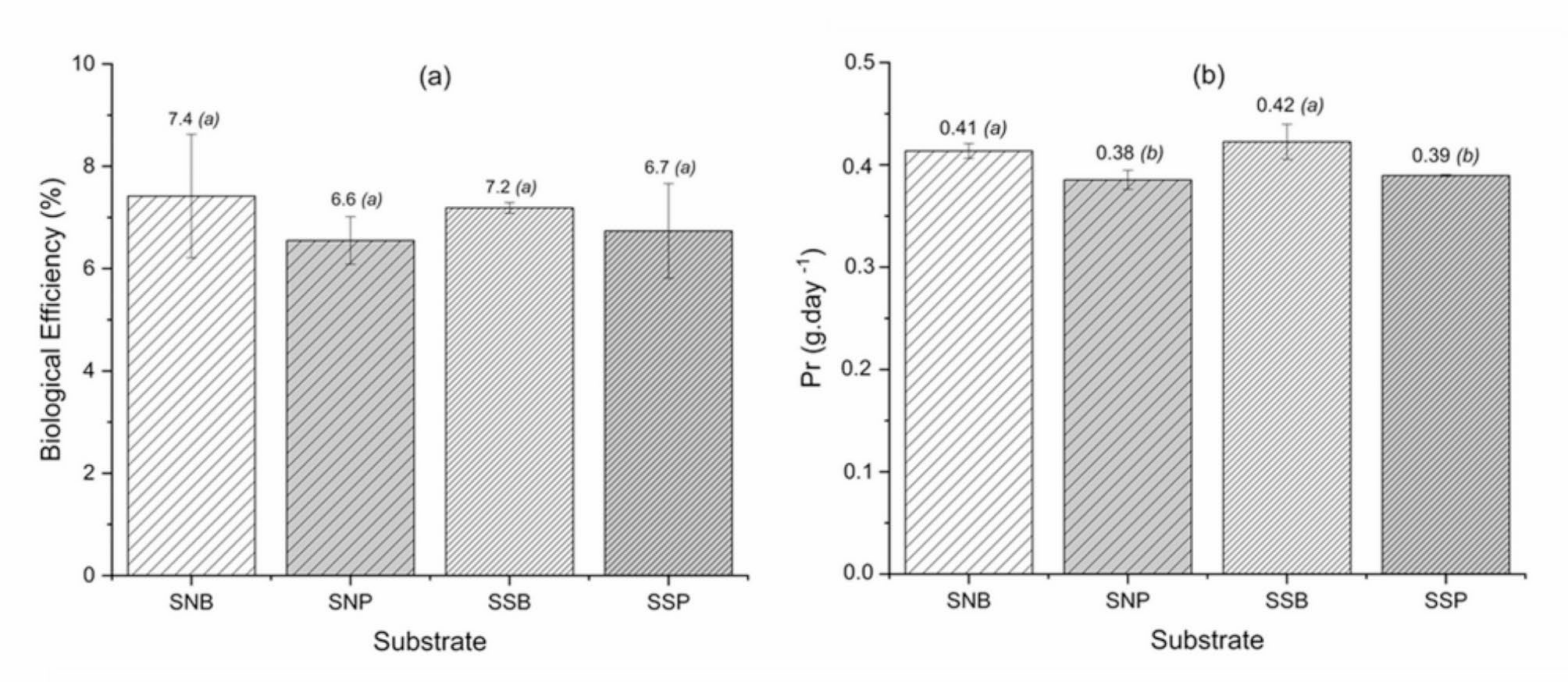
Values (means ± standard deviations) of biological efficiency (BE - %) (**A**) and productivity (Pr - g day^−1^) (**B**) for *P. sajor-caju* production. SNB = natural brewer’s spent grains + banana leaves, SNP = natural brewer’s spent grains + peach palm leaves, SSB = dried brewer’s spent grains + banana leaves, SSP = dried brewer’s spent grains + peach palm leaves. Equivalent letters indicate no significant difference between substrates (Tukey test, 5% significance).

The observed variation in different parameters found in the brewer’s spent grain used in this study and the values reported in the literature can be attributed to several factors, including the barley cultivar, harvest season, malt grinding conditions, and the quality and type of adjuvants (corn, rice, wheat, and sorghum) added during the process. The brewing process also determines the chemical composition of these byproducts^3,5^. The results of the brewer’s spent grain characterization (Table 2) indicate that the SN and SS substrates provide sufficient nutritional conditions for the growth of fungi of the genus *Pleurotus*, which are characterized as fungi with low nutritional requirements^38,44^. However, the experiments with 100% brewer’s spent grains SN and SS did not produce mushrooms, likely due to the compaction of the brewer’s spent grain after sterilization, which hindered mycelial growth. The compaction hinders gas exchange in the substrate because it is related to the granulometry and nature of the substrate, which interferes with the oxygenation and development of the mushrooms^44^. Consequently, it was determined that the residue of brewer’s spent grain should be combined with two distinct agro-industrial byproducts that are widely available in the northern region of Santa Catarina, Brazil: banana leaves^6^ and peach palm leaves^7^. It is important to note that despite the C: N ratios reported in the literature for banana leaves (41.1:1) and peach palm leaves (20.6:1) being nearly double that of each other, these values are sufficient for the cultivation of *Pleurotus*^44^. This explains why there was no significant difference in the biological efficiency (%) of the process using one or the other substrate (Fig. 2A). However, the substrates utilizing banana leaves exhibited higher values of productivity than those utilizing peach palm leaves (Fig. 2B). This difference can be attributed to the higher fiber content of peach palm leaves^7^, which impedes enzymatic activity, prolonging the maturation period of the mushrooms.

There was no statistically significant difference between the productivity of the two substrates, fresh and dried brewer’s spent grain (Fig. 2B). Consequently, the substrate selected for *Pleurotus sajor-caju* production was natural brewer’s spent grains mixed with banana leaves (SNB), as this substrate can be used shortly after being generated in industry. The values of BE and Pr found in this study were greater than those reported by other researchers using the *Pleurotus* genus and peach palm leaves exclusively as substrates^6,7,28^.

### Nutritional qualities of the mushrooms

Table 3 presents the composition of *Pleurotus sajor-caju* mushrooms cultivated in natural brewer’s spent grains mixed with banana leaves (SNB) on a dry and wet basis in relation to carbohydrates, lipids, fibers, proteins, phosphorus, potassium, and sodium. These values were compared to those established in Decree No. 54^45^, issued by the Brazilian National Agency for Sanitary Surveillance (ANVISA), which approves the Technical Norms on Complementary Nutritional Information. Table 3 indicates that *Pleurotus sajor-caju* produced in this study can be considered a food that contains sugars, has a very low sodium content and is a source of phosphorus. As evidenced by the literature, mushrooms are low in fat, high in fiber, and high in protein, phosphorus, potassium, and other nutrients^8,9^. However, due to their capacity to bioaccumulate heavy metals from the soil, they have also been studied for use in soil bioremediation processes^46,47^.

**Table 3:** Mean carbohydrate, total fat, fiber, protein, phosphorus, potassium, and sodium values for the *P. sajor caju* mushroom and comparison with Decree no. 54 values^45^.

Nutrients	*P*. sajor-caju dry	Conclusion of according to Decree no. 54*	*P*. sajor-caju wet	Conclusion of according to Decree no. 54*
Carbohydrate (g·100 g^−1^)	54.6	−	6.3	-
Total fat (g·100 g^−1^)	2.0	Low content	0.2	Not containing
Fiber (g·100 g^−1^)	6.2	High content	0.7	Not a source
Proteins (g·100 g^−1^)	30.9	High content	3.3	Not a source
Phosphorus (mg·100 g^−1^)	1273.9	High content	137.6	Source
Potassium (mg·100 g^−1^)	1864.8	High content	201.4	Not a source
Sodium (mg·100 g^−1^)	103.3	Low content	11.2	Very low content

*Total fat – Low content: Maximum of 3 g·100 g^−1^. Not containing: Maximum of 0.5 g·100 g^−1^. Fibers – Source: Minimum of 3 g·100 g^−1^. High content: Minimum of 6 g·100 g^−1^. Proteins – Source: Minimum of 10% of dietary reference intake (DRI) of reference.100 g^−1^. High content: Minimum DRI of 20% relative to the reference value of 100 g^−1^. DRI = 50 g. Na – Low content: Maximum of 120 mg·100 g^−1^. Very low content: Maximum of 40 mg·100 g^−1^. Not containing: Maximum of 5 mg·100 g^−1^. Minerals – Source: Minimum of 15% of the DRI of reference.100 g^−1^. High content: A minimum of 30% of the DRI of reference.100 g^[− 145^. DRI *P* = 700 mg^48^. DRI K = 4700 mg^49^.

In order to ensure that they are suitable for consumption, it is necessary to perform a heavy metal analysis to determine the levels of Pb and Hg in their fruiting bodies (mushrooms)^50^. Consequently, further characterization of *P. sajor-caju* mushrooms was conducted in terms of heavy metal content (mg kg^−1^). The Pb and Hg contents in mushrooms on a dry and wet basis are below the maximum limits permitted, as set forth in Decree No. 88^50^, which regulates the maximum tolerable limits (LMTs) of inorganic contaminants in foods (Table 4). Some studies have evaluated the content of heavy metals in mushrooms^7,51–54^. The typical elements that accumulate in macrofungi are Au, Ag, As, Br, Cd, Cs, Cu, Hg, Rb, Se, V, Zn, and Cl. In contrast, Co, Cr, F, I, Ni, Sb, Sn, Th, U, and rare earth elements are typically found in low concentrations. The highest concentrations of these elements are typically found in the cap of the mushroom. A study conducted in various regions of Poland revealed that the consumption of mushrooms may result in a significant exceeding of the recommended daily intake (RDI) for Cd and Cu. In a study conducted in the Athens metropolitan area, an intriguing novel finding was presented. No significant correlations were detected between the total concentrations of heavy metals in soils and those in mushrooms. However, the concentrations of K, Na, and P in soils were found to be associated with the contents of several metals in fruit bodies. In particular, Pb and Cu were found to exceed the recommended dietary allowances or tolerable upper intake levels.

**Table 4:** Levels of pb and hg in mg kg^−1^ of mushrooms on dry and wet basis and comparison with the maximum tolerance limits of inorganic contaminants in food by Decree No.88^50^.

Nutrients	*P*. sajor-caju (dry)	*P*. sajor-caju (wet)	Maximum tolerance limit (Decree No. 88)
Pb (mg kg^−1^)	0.0256	0.002	0.80
Hg (mg kg^−1^)	< 0.0007	< 0.0007	0.00

### Mycelium-based composite production

**Fig. 3:**
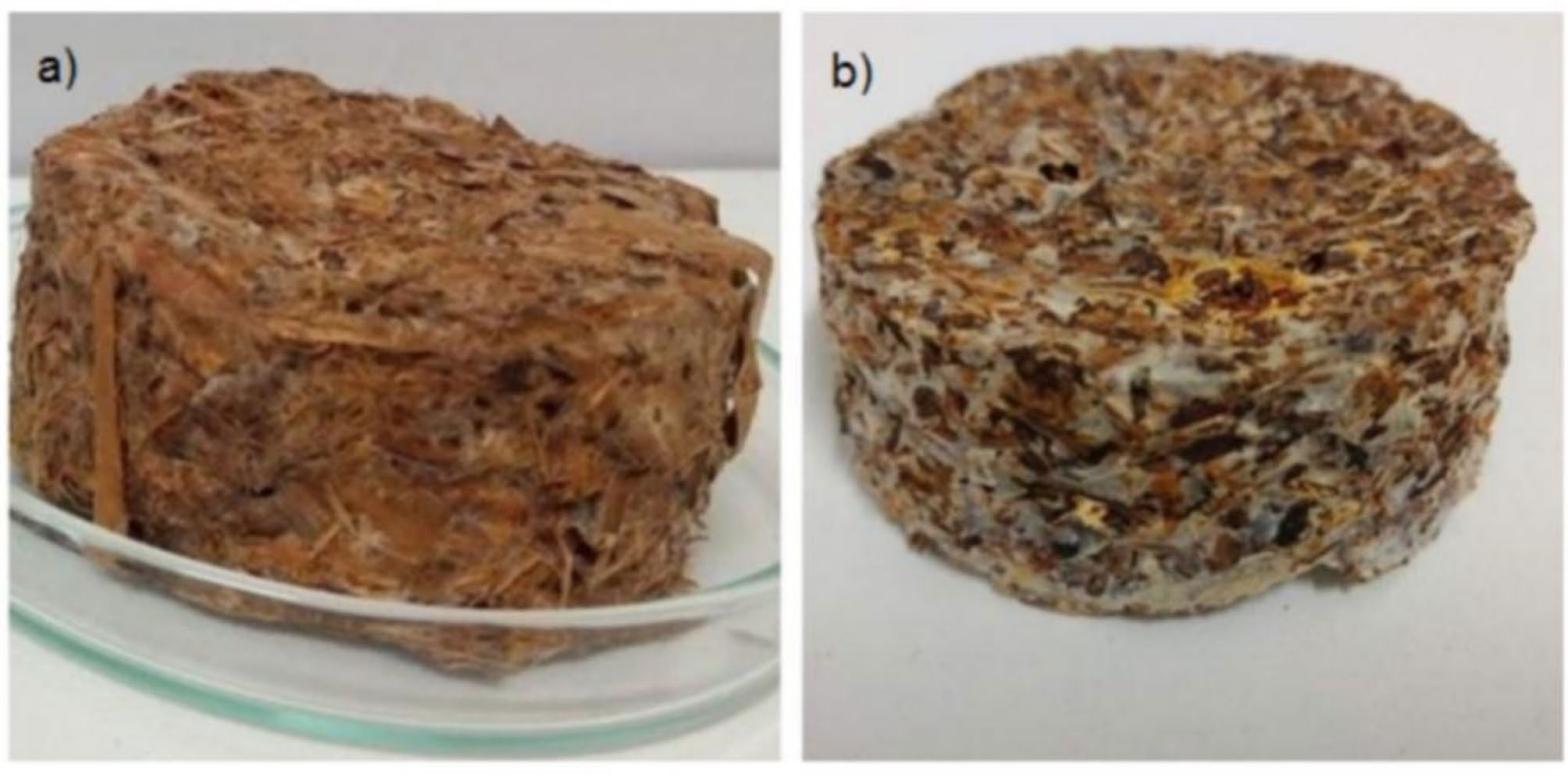
Mycelium-based composites produced with SNB fresh (**a**) and SNB residual (**b**) substrates.

The natural brewer’s spent grain mixed with banana leaves (SNB) was selected as the material for the production of mycelium-based composites. The mycelium-based composites produced with SNB residual substrate exhibited a significantly greater *Pleurotus sajor-caju* mycelial mass than that observed in the mycelium-based composites produced with SNB fresh substrate (Fig. 3). This can be attributed to the higher protein and ash content of the SNB residual substrate, which provides a superior nutritional profile for the *P. sajor-caju* cultivation, This can be attributed to the higher protein and ash content of the SNB residual substrate, which provides a superior nutritional profile for the *P. sajor-caju* culture, This can be attributed to the higher protein and ash content of the SNB residual substrate, which provides a superior nutrient profile for the *P. sajor-caju* culture, and consequently a higher biomass density, with bonds of varying strength, resulting in the formation of a protective layer or “fungal skin” around the exterior of the substrate, as the hyphae are able to both penetrate and envelop the substrate, improving the mechanical properties of the mycelium-based composites. The degree of colonization, the thickness of the skin (fungal skin), and the type of biomass, in turn, influence the stiffness and water resistance of the resulting materials, as will be demonstrated in the next set of results from this study^55^. Despite the greater apparent mycelial mass, the mycelium-based composites produced with SNB residual substrate exhibited a longer total process time (21 days) than the mycelium-based composites produced with SNB fresh substrate (16 days). The global process time (total process time + drying time) exhibited a similar trend, with the mycelium-based composites produced with SNB residual substrate (23.2 days) exhibiting approximately 5 days longer than those produced with SNB fresh substrate (18 days). The total processing time of mycelium-based composites in the literature varies from 12 to 45 days and depends on the interaction between the fungus and the substrate used. One of the most frequently utilized fungi for the production of mycelial-based composites is the *Pleurotus* genus. With regard to the substrates, a diverse range of lignocellulosic wastes can be employed; but generally are used those that are more disposable in the region of mycelium-based composites production^16,55,56^.

### Physical and mechanical properties of mycelium-based composites

For satisfactory growth of mycelia of the genus *Pleurotus*, the substrate should be approximately 70% moisture^15,57^. The initial moisture content of both substrates (SNB fresh and SNB residual) did not differ significantly, with an average of 78.5%. The values reported in the literature for initial moisture content ranged from 59.7 to 83.2%, using different substrates (sawdust, straw, cotton, palm sugar fiber, and cassava bagasse) to produce mycelium-based composites^21,56,58^ After the fungus has been grown, the composite material is removed from the mold and subjected to one of three methods of dehydration: hot pressing, oven drying, or air drying. The objective of these processes is to neutralize the fungus and reduce the final moisture content of the mycelium-based composite. The specific drying method employed depends on the substrate utilized, with hot pressing and oven drying being favored by industry due to their efficiency. Hot pressing also has the additional benefit of consolidating and densifying the material, which results in higher mechanical properties. In this study an oven was used to dehydrated the mycelium-based composites. The final moisture contents obtained for both substrates (SNB fresh and SNB residual) did not differ significantly, with an average of final moisture content of 5.4%. The values reported in the literature for final moisture content ranged from 5.8 to 9.6%^21,56,58^. The humidity at which no fungus can grow is less than 14%. Nevertheless, a slight increase in humidity to 15 to 16% will permit the growth of the stress-tolerant fungus *Aspergillus* spp. The mycelium-based composite humidity found in this study for the final product is less than 14%, a value necessary to inhibit fungal growth, making the product viable and functional^14–16^.

**Fig. 4:**
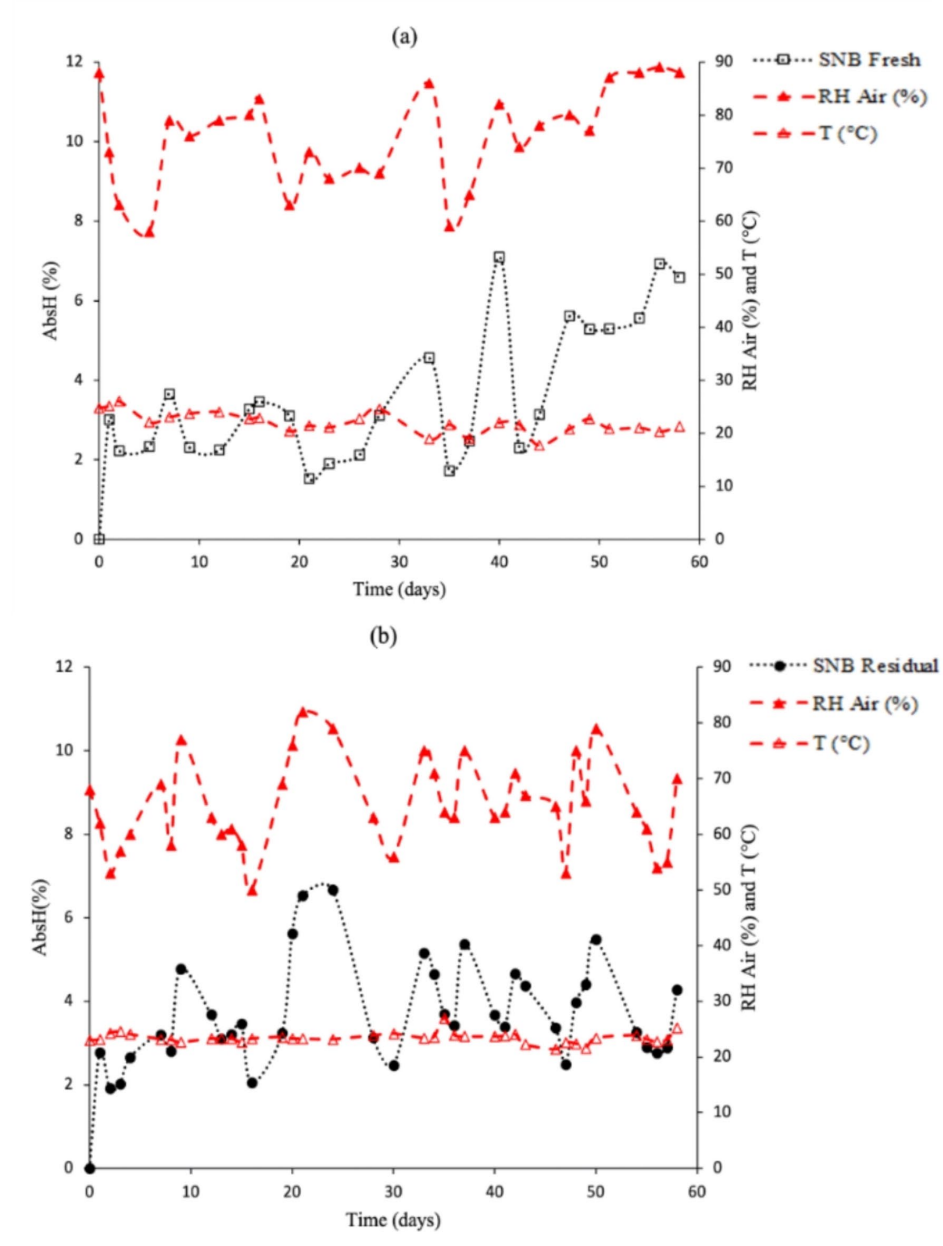
Influence of air humidity (AbsUar %) on mycelium-based composites produced with SNB fresh and SNB residual substrates, during 60 days of exposure. Dashed lines with red symbols (▲, ∆) indicate relative air humidity (RH air - %) and room temperature (T - °C) at the time of weighing.

Figure 4 illustrates the moisture sorption of mycelium-based composites obtained in this study. The behavior of the mycelium-based composites produced with SNB fresh and SNB residual substrates was found to be very similar with respect to moisture sorption test. All the proof bodies were influenced by the air relative humidity, with the percentage of moisture sorption increasing as the relative humidity increased and decreasing when it decreased; this phenomenon has also been observed by other authors. The mycelium-based composites produced with SNB fresh substrate exhibited a maximum sorption of 7.1% (82% RH and 22 °C), while the mycelium-based composites produced with SNB residual substrate exhibited a maximum of 6.6% (79% URar and 23.2 °C). The purpose of this analysis is to simulate the exposure of the product to environmental conditions. The majority of fungi are considered to be hydrophobic, a property that is related to hydrophobins, which are low-molecular-weight proteins that are found in the cell wall of fungi only. The water sorption is highly influenced by the mycelial matrix, while the type of substrate exerts a less significant influence^19,55^. The presence of mycelia hyphae in the mycelium-based composites serves as a hydrophobic reinforcement in the material structure. Air humidity sorption is a property of significance in determining the quality and durability of the final product^17,56,59^. The values obtained in this study are lower than those reported in other studies of mycelium-based composites (water sorption of 10 to 32% at a relative humidity of 75 to 90%)^16,17,19,59^.

**Fig. 5:**
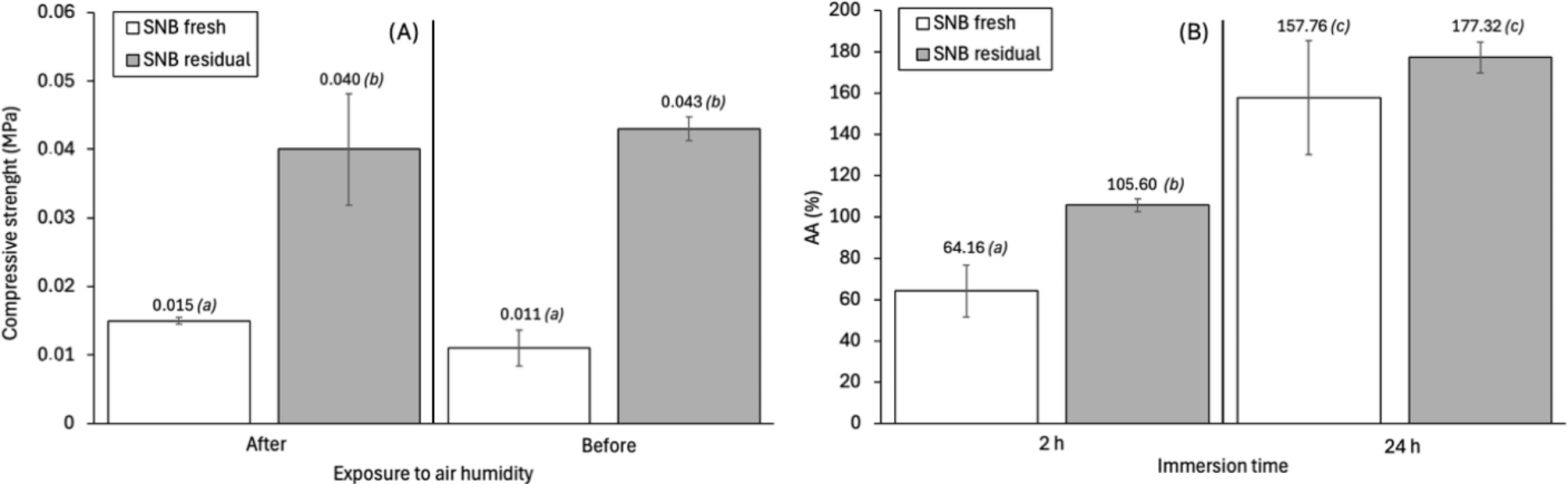
(**A**) Water sorption values (AA% - means ± standard deviations) and (**B**) compressive strength values (means ± standard deviations) of the mycelium-based composites produced with the SNB fresh and SNB residual substrates. Comparison of results for mycelium-based composites using the two substrates: equal letters indicate no significant difference (Tukey test, 5% significance).

Figure 5A illustrates the water sorption of mycelium-based composites produced with SNB fresh and SNB residual substrates. The mycelium-based composite produced with SNB residual substrate exhibited a significantly higher water sorption value (105.6%) after 2 h of immersion compared to the mycelium-based composite produced with SNB fresh substrate (64.16%). This difference can be attributed to the higher mass of the mycelium-based composite produced with SNB residual substrate, which results in greater water sorption. However, after 24 h of immersion, the two mycelium-based composites exhibited no statistically significant difference in the total amount of water absorbed (mean 167%). One significant challenge for the utilization of mycelium-based composites is their propensity to absorb considerable quantities of water. Consequently, to mitigate water absorption by a material, the density of the material must be increased, or the outer surface must be rendered hydrophobic^19,21,58^.

Figure 5B illustrates the compressive strength results for mycelium-based composites produced with SNB fresh and SNB residual substrates before and after 60 days of exposure to air humidity. The mycelium-based composites produced with SNB residual substrate exhibited the highest compressive strength, approximately 0.04 MPa, which was approximately 2.7 times greater than those of the mycelium-based composites produced with SNB fresh substrate (0.015 MPa). This significant increase in compressive strength highlights the influence of utilizing SNB residual substrate on the mechanical properties of mycelium-based composites produced. Additionally, the results highlight the potential of utilizing the same mixture of residues to produce two distinct, high-value products in a sequential process. The SNB fresh substrate exhibited greater heterogeneity due to the presence of banana leaf fibers, which made it more difficult to bond the material, which had many empty spaces, reducing the density (164 kg m^− 3^). In contrast, the SNB residual substrate exhibited greater homogeneity in terms of particle size, as it had been previously degraded by fungi, which may have promoted a greater density (2–42 kg m^−3^). The density values demonstrate a significant increase in the mycelium-based composites produced with SNB residual substrate, with a 47% increase in comparison to the composites produced with SNB fresh substrate. After 60 days of exposure to air humidity, the compressive strength of both mycelium-based composites remained relatively stable (Fig. 5B). This indicates that the material retained its resistance to compression for a period of 60 days.

The density of mycelium-based composites varies considerably depending on the substrate utilized. Mycelium-based composites containing agricultural byproducts, such as fibers and straw, have a lower density (60–130 kg m^−3^) than mycelium-based composites containing forest byproducts, such as sawdust (87–300 kg m^−3^)^15^. The density of mycelium alone varies from 30 to 50 kg m^−3^, while the density of natural fibers varies from 1200 to 1500 kg m^−3^, and the density of wood varies from 300 to 880 kg m^−3^. This implies that the incorporation of mycelia into these materials results in a reduction in the composite density^22,59^.

A comparative analysis of the mechanical properties of several mycelium-based composites, produced using different substrates and fungi, revealed a range of values for density and compression strength, from 59 to 552 kg m^−3^ and from 0.17 to 4.44 MPa, respectively. Comparing these values with those of materials commonly used in packing and construction activities, such as synthetic foams and wood products, with regard to density the mycelium-based composites exhibit values for density that are higher than those of synthetic foams but lower than those of wood products. The density of synthetic foams ranges from 11 to 50 kg m^− 3^ and from 30 to 100 kg m^−3^ for polystyrene and polyurethane, respectively. The density of wood products ranges from 460 to 680 kg m^− 3^ and from 850 to 1030 kg m^−3^ for plywood and hardwood, respectively^15,55^. The density of mycelium-based composites is also lighter than other wood-based composites, including medium-density fiberboard (500–1000 kg m^−3^) and oriented strandboard (550–700 kg m^−3^)^55,59^. Consequently, mycelium-based composites are sufficiently lightweight to be employed in the packaging of food and household appliances. With regard to compression strength, the synthetic foams present values ranging from 0.03 to 0.69 MPa and from 0.002 to 48 MPa for polystyrene and polyurethane, respectively. The density of the wood products ranges from 460 to 680 kg m^−3^ and from 850 to 1030 kg m^−3^ for plywood and hardwood, respectively. The mycelium-based composites exhibit lower values of compression strength than wood products and polyurethane, and a higher mean value of compression strength than polystyrene. The compressive strength of materials is a critical parameter in determining their suitability for use in packaging applications, for example. Another desirable property for materials used in packaging is low density. A higher compressive strength indicates a reduced probability of damage to the product when a substantial load is applied^15,21,58^. In Brazil, expanded polystyrene (EPS) is a common packaging material with a density between 13 kg m^−3^ and 30 kg m^−3^. Its compressive strength is 0.035 MPa, which is comparable to the values obtained in this study (0.04 and 0.043 MPa). The compressive strength values obtained by other researchers in similar studies ranged from 0.015 to 2.00 MPa^16,17,19^. The mycelium-based composite produced with the substrate coconut powder-based, supplemented with wheat bran and the edible mushroom “Shiitake” (*Lentinula edodes*), exhibited compressive strength values ranging from 0.05 to 0.10 MPa. These findings suggest that the biocomposite may serve as an ecological alternative in the production of packaging materials due to its mechanical properties. To evaluate the commercial use of composites, it is necessary to consider the compressive strength at 10% maximum deformation. The expanded polystyrene (EPS) values can range from 0.033 to 0.165 MPa, depending on the specific type of isopor^16^. The mycelium-based composites produced with the substrates cotton stalk, wheat bran, and carbonate sand and the strains *Pleurotus ostreatus*, *Oudemansiella radicata*, and *Acremonium* sp. exhibited compressive strength of approximately 0.51 MPa and a density of 310–400 kg m³. These properties satisfy the requirements for use as backfill materials in geotechnical engineering^17^. The mycelium-based composites produced using *Pleurotus ostreatus* and two substrates oat husk and rapeseed cake after oil pressing exhibited compressive strength of 0.015 MPa and 0.3 MPa, respectively, and a density of 38 kg m^−2^ and 50 kg m^−2^, respectively. According to the authors, the mycelium-based composites have high potential and ability to be more widely used in future material solutions due to their sustainability, low cost, low energy consumption during processing, and ease of production^19^. In terms of the costs associated with the production of mycelium-based composites, the literature indicates that the price ($US/kg) ranges from 20 to 80 times less than that of synthetic foams (polystyrene and polyurethane, respectively) and from 10 to 40 times less than that of wood products (plywood and hardwood, respectively)^15,55^.

### Biodegradability

**Fig. 6:**
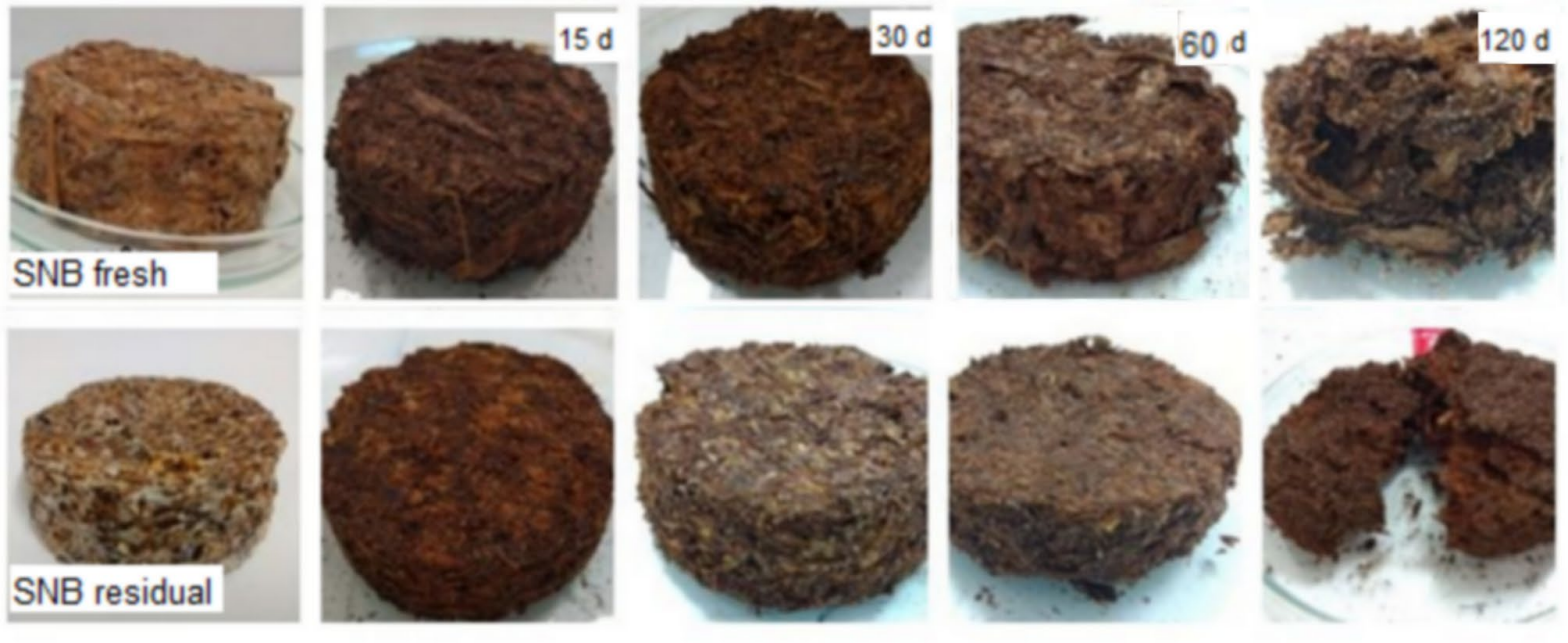
Biodegradability of mycelium-based composites produced with SNB fresh and SNB residual substrates after 15, 30, 60, and 120 days of testing.

Biodegradability analysis was conducted visually, as it was not feasible to quantify the material gravimetrically. The mycelium-based composites produced with SNB fresh and SNB residual substrates were excavated after 15, 30, 60, and 120 days of testing (Fig. 6). The mycelium-based composites produced with SNB fresh substrate disintegrated more readily than the mycelium-based composite produced with SNB residual substrate. The most significant disintegration occurred after 60 days of experimentation. The greater the compaction and the higher the mycelial density are, the longer the disintegration period. In the context of biodegradation, the mycelium binder tends to break down first, while the loose fibers remain. The fibers do not entirely disintegrate but rather darken and decrease in size^60^.

Despite the 120-day exposure period, it was not possible to identify complete disintegration of the material in either mycelium-based composites. While complete disintegration was not observed, this material degrades more readily than other materials used in packaging, such as expanded polystyrene (EPS), which is 100% recyclable but does not degrade easily. In a study involving *Ganoderma resinaceum* and hemp, the biodegradability in soil was assessed, revealing a mass reduction of 43% for inert samples and 50.8% for live samples after 16 weeks of burial in potting soil^60^. In other studies, similar findings were observed: the mycelium-based composites disintegrated in soil, thus reinforcing the claim of their biodegradability. The drying process halts decomposition, thus enabling the conservation of fungal composites under environmental conditions. It is evident that mycelium-based composites cannot be employed for long-term materials due to their diminishing resistance to water, humidity, and mold growth. Upon rehydration, these composites can serve as nutrient and energy sources for soil saprophytic microorganisms, thereby promoting nutrient cycling and reducing the environmental persistence of biopackaging^16,22,57,60^.

## Conclusions

The utilization of brewer’s spent grains mixed with banana leaves showed high potential for the production of *Pleurotus sajor-caju* (biological efficiency of 7.4 ± 1.2% and productivity of 0.41 ± 0.07 g day^−1^) as well as for the mycelium-based composites, especially when the residual substrate (after mushroom harvesting) was used. The mushrooms produced have a high nutritional value, containing sugars, low fat, low sodium, high fiber, high protein, high phosphorus, and high potassium. The mycelium-based composites, especially those produced with the residual substrate, exhibited increased mycelium density, resulting in higher compressive strength (0.04 MPa) and apparent density (242 kg m^−3^). The results demonstrate the synergistic effect of combining the two approaches under investigation. The use of brewer’s spent grains mixture with banana and beach palm leaves improved the production instead of using only the leaves as substrate. The findings contribute considerably to the scientific community’s efforts to emphasize the necessity of transitioning from a traditional, linear economy to a circular one. This transition involves addressing concerns related to the rational use of natural resources, waste valorization, and prolonging the life cycle of materials, thereby promoting sustainability for future generations.

In future studies, it would be beneficial to characterize mycelium-based composites in terms of impact resistance, tensile and flexural strength, thermal conductivity, acoustic absorption, and flame resistance. This would assist in identifying the most suitable further applications. Additionally, the new mixtures of banana leaves and peach palm leaves with brewer’s spent grains should be evaluated; as well as, the potential of *Pleurotus sajor-caju* as an antimicrobial agent for the development of active and intelligent packaging should be investigated. Furthermore, the environmental footprint of mycelium-based composites must be evaluated in comparison to synthetic materials, such as life cycle analysis (LCA) - energy-efficient manufacturing process, no harmful effects during the life cycle, how to manage the degradation of biocomposites and the resulting waste; as well as the costs associated with the manufacturing process - the cost of raw materials, transportation of residues, and other relevant expenses, must also be assessed.

## Data Availability

All data generated or analyzed during this study are included in this manuscript.
